# Association between variables measured in the ambulance and in-hospital mortality among adult patients with and without infection: a prospective cohort study

**DOI:** 10.1186/s12873-022-00746-x

**Published:** 2022-11-23

**Authors:** Ulrika Margareta Wallgren, Hans Järnbert-Pettersson, Jan Sjölin, Lisa Kurland

**Affiliations:** 1 Department of Clinical Science and Education, Karolinska Institutet, Sjukhusbacken 10, 118 83 SöderssjukhusetStockholm, Sweden; 2 Fisksätra Vårdcentral (Primary Health Care Center), Fisksätra Torg 20, 133 41 Saltsjöbaden, Sweden; 3 Department of Medical Sciences, Örebro University, Campus USÖ, Södra Grev Rosengatan 32, 701 12 Örebro, Sweden; 4 Department of Medical Sciences, Uppsala University, Akademiska Sjukhuset, 751 85 Uppsala, Sweden

**Keywords:** Mortality, Infection, Sepsis, Emergency medical services, Prehospital, Emergency care

## Abstract

**Background:**

Patients presenting with infection to the ambulance are common, but risk factors for poor outcome are not known. The primary aim of the current study was to study the association between variables measured in the ambulance and mortality among adult patients with and without infection. The secondary aim was to study the association between these variables and mortality in a subgroup of patients who developed sepsis within 36 h.

**Methods:**

Prospective cohort study of 553 ambulance patients with, and 318 patients without infection, performed in Stockholm during 2017–2018. The association between 21 variables (8 keywords related to medical history, 6 vital signs, 4 blood tests, and age, gender, comorbidity) and in-hospital mortality was analysed using logistic regression.

**Results:**

Among patients with infection, inability of the patient to answer questions relating to certain symptoms such as pain and gastrointestinal symptoms was significantly associated with mortality in univariable analysis, in addition to oxygen saturation < 94%, heart rate > 110 /min, Glasgow Coma Scale (GCS) < 15, soluble urokinase Plasminogen Activator Receptor (suPAR) 4.0–7.9 ng/mL, suPAR ≥ 8.0 ng/mL and a Charlson comorbidity score ≥ 5. suPAR ≥ 8.0 ng/mL remained significant in multivariable analysis (OR 25.4; 95% CI, 3.2–199.8). Among patients without infection, suPAR ≥ 8.0 ng/mL and a Charlson comorbidity score ≥ 5 were significantly associated with mortality in univariable analysis, while suPAR ≥ 8.0 ng/mL remained significant in multivariable analysis (OR 56.1; 95% CI, 4.5–700.0). Among patients who developed sepsis, inability to answer questions relating to pain remained significant in multivariable analysis (OR 13.2; 95% CI, 2.2–78.9), in addition to suPAR ≥ 8.0 ng/mL (OR 16.1; 95% CI, 2.0–128.6).

**Conclusions:**

suPAR ≥ 8.0 ng/mL was associated with mortality in patients presenting to the ambulance both with and without infection and in those who developed sepsis. Furthermore, the inability of the ambulance patient with an infection to answer questions relating to specific symptoms was associated with a surprisingly high mortality. These results suggest that suPAR and medical history are valuable tools with which to identify patients at risk of poor outcome in the ambulance and could potentially signal the need of enhanced attention.

**Trial registration:**

ClinicalTrials.gov, NCT03249597. Registered 15 August 2017—Retrospectively registered, https://clinicaltrials.gov/ct2/show/NCT03249597.

## Background

Patients presenting to the ambulance with an infection are common. It is, however, not known how to identify those at risk of a poor outcome. Although age, comorbidity, vital signs, and male sex have been identified as risk factors for death among emergency department (ED) patients with an infection [1–5], less is known about these risk factors in the ambulance setting. Patients presenting to the ambulance are on average older, have higher triage levels and higher mortality rates [6–8]. Hence, it is important to investigate risk factors for poor outcome in this population.

Vital signs are used as the cornerstone of most triage and monitoring systems to identify level of acuity based on the premise that they are associated with mortality [9, 10]. However, vital signs alone are insufficient to predict mortality among patients with an infection since one third of the patients with infection present with normal vital signs in the ambulance [11]. Hence, variables such as medical history may need to be considered to identify patients with poor outcome.

We hypothesized that it is important to consider variables other than vital signs when aiming to identify patients with poor outcome among those presenting to the ambulance with an infection. The primary aim was to study the association between variables measured in the ambulance and in-hospital mortality among adult ambulance patients with and without infection. The secondary aim was to study the association between these variables and mortality in the patients who developed sepsis within 36 h from ED arrival.

## Methods

### Study design and setting

The current study was based on the prospective cohort study of 871 adult, non-trauma, ambulance patients with and without infection transported by ambulance to hospitals in Stockholm, during the period of April 3rd, 2017, and August 30th, 2018; the Predict Sepsis study [12] (Clinical Trials identifier NCT03249597). All patients were enrolled by the ambulance personnel and transported by the ambulance provider Samariten Ambulans AB [13] to one of the seven major hospital EDs (Södersjukhuset, Karolinska Huddinge, Karolinska Solna, St Göran, Danderyd, Norrtälje, Södertälje) in Stockholm County Council [12]. All ambulances were staffed with at least one nurse specialist and one emergency medical technician [14].

The association between 21 variables measured in the ambulance and in-hospital mortality was analysed in the two ambulance groups, i.e., with and without infection, and in the subgroup of patients who developed sepsis within 36 h from ED arrival.

### Selection of study participants

#### Inclusion criteria

Inclusion criteria were adult (≥ 18 years) non-trauma patients with and without infection according to clinical judgment by the ambulance personnel.

#### Exclusion criteria

Exclusion criteria were: 1) lack of written consent; 2) trauma other than fall at home; 3) patient leaving ED prior to physician assessment; 4) direct admission to geriatric hospital i.e., bypassing the ED; 5) missing hospital records, 6) missing personal identification number and 7) missing categorization with respect to infection or not upon inclusion.

### Data collection and handling

#### Keywords related to medical history

Eight keywords related to medical history were registered in the study protocol in the ambulance. The keywords were derived from a prior study of septic patients in the ambulance [15] and were all reflective of current medical history, predominantly symptom presentation, which exceeded a prevalence of 20%. These eight keywords were: “fever or suspected fever”, “pain”, “acute altered mental status”, “weakness of the legs”, “breathing difficulties”, “loss of energy”, “gastrointestinal symptoms” and “risk factors for sepsis” [12], see Table 1. All symptoms were required to have a new-onset (within days) or to be aggravated. There were three categories for each keyword; “yes”/ “no” / “inability to answer”. “Inability to answer” was chosen if the patient could not answer or did not know whether he/she experienced the specific keyword. Data was recorded as missing when no category was chosen. Questions relating to the keywords were primarily answered by the patient. However, if the patient was not able to answer him/herself, relatives/bystanders and ambulance personnel were allowed to document their observations with respect to these questions.

**Table 1:** Characteristics of 871 ambulance patients with and without infection according to clinical judgment by ambulance personnel

**Variable**	**Patients with infection**		**Patients without infection**		
	**Number (%) *n* = *553***	**Median (IQR)**	**Number (%) *n* = *318***	**Median (IQR)**	P-value^*^
**Age** (years)		78 (71–85)		74 (59–83)	** < 0.001**
**Gender**					0.054
-male	331 (59.9)		169 (53.1)		
**Ambulance priority**					** < 0.001**
(n**)	547		313		
1	100 (18.3)		19 (6.1)		
2	386 (70.6)		246 (78.6)		
3	61 (11.2)		48 (15.3)		
**Keywords related to medical history**					
1. Fever or suspected fever					** < 0.001**
(n**)	552		316		
yes	404 (73.2)		31 (9.8)		
no	134 (24.3)		279 (88.3)		
inability to answer	14 (2.5)		6 (1.9)		
2. Pain					**0.017**
(n**)	551		315		
yes	256 (46.5)		145 (46.0)		
no	274 (49.7)		168 (53.3)		
inability to answer	21 (3.8)		2 (0.6)		
3. Acute altered mental status					** < 0.001**
(n**)	552		316		
yes	328 (59.4)		70 (22.2)		
no	208 (37.7)		242 (76.6)		
inability to answer	16 (2.9)		4 (1.2)		
4. Weakness of the legs***					** < 0.001**
(n**)	552		316		
yes	420 (76.1)		105 (33.2)		
no	109 (19.7)		205 (64.9)		
inability to answer	23 (4.2)		6 (1.9)		
5. Breathing difficulties					** < 0.001**
(n**)	550		315		
yes	280 (50.9)		70 (22.2)		
no	251 (45.6)		240 (76.2)		
inability to answer	19 (3.4)		5 (1.6)		
6. Loss of energy					** < 0.001**
(n**)	552		315		
yes	491 (88.9)		162 (51.4)		
no	50 (9.1)		148 (47.0)		
inability to answer	11 (2.0)		5 (1.6)		
7. Gastrointestinal symptoms:vomiting/diarrhoea					** < 0.001**
(n**)	551		316		
yes	188 (34.1)		67 (21.2)		
no	337 (61.2)		247 (78.2)		
inability to answer	26 (4.7)		2 (0.6)		
8. Risk factors for sepsis****					** < 0.001**
(n**)	550		315		
yes	229 (41.6)		33 (10.5)		
no	294 (53.4)		275 (87.3)		
inability to answer	27 (4.9)		7 (2.2)		
**Vital parameters**					
1. Respiratory rate (min^−1^)		21 (18–28)		16 (15–19)	** < 0.001**
2. Oxygen saturation (%)		94 (91–97)		97 (95–99)	** < 0.001**
3. Heart rate (min^−1^)		94 (80–109)		80 (70–95)	** < 0.001**
4. Systolic BP (mmHg)		135 (120–150)		140 (124–160)	** < 0.001**
5. GCS (score)		15 (15–15)		15 (15–15)	** < 0.001**^**α**^
6. Temperature (°C)		38.3 (37.5–39.1)		36.8 (36.5–37.1)	** < 0.001**
**Blood tests**					
1. P-Glucose (mmol/L)		7.8 (6.8–9.7)		7.1 (6.1–8.6)	** < 0.001**
2. P-Lactate (mmol/L)		1.7 (1.3–2.6)		1.6 (1.2–2.4)	0.103
3. P-suPAR (ng/mL)		4.8 (3.5–6.7)		3.5 (2.5–4.7)	** < 0.001**
4. P-HBP (ng/mL)		12.9 (5.9–28.4)		5.9 (5.9–9.7)	** < 0.001**
**Comorbidity**					
Charlson comorbidity score		2 (1–4)		1 (0–2)	** < 0.001**
**Admitted to in-hospital care**	455 (82.3)		179 (56.3)		** < 0.001**
**Sepsis within 36 h**	230/551 (41.7)		12/317 (3.8)		** < 0.001**
**In-hospital mortality**	33 (6.0)		8 (2.5)		**0.021**

IQR = Interquartile range, min = minute, BP = Blood Pressure, GCS = Glasgow Coma Scale, °C = degrees Celsius, suPAR = soluble urokinase Plasminogen Activating Receptor, HBP = Heparin Binding Protein, ED = Emergency Department, qSOFA = quick SOFA (Sequential Organ Failure Assessment score), ICD = International Statistical Classification of Diseases and Related Health Problems

^*^Chi2 test was used to compare proportions and Mann–Whitney U to compare medians between the groups

^**^number of patients with documentation of the variable

^***^difficulties to walk/stand/raise/fallen/found on the floor or similar

^****^such as infection/antibiotic treatment/chemotherapy/ surgical/urological procedure/new blood-/urinary catheters last weeks or alcohol/drug abuse

^α^The p-value is significant despite medians and ICR being equal which is explained by the fact that > 75% of the patients in both groups presented with GCS 15 but the distribution of lower GCS scores differed between the groups, i.e., lower GCS scores were more frequent in the group with infection

Bold numbers indicate significant P-values

### Vital signs

The first value measured in the ambulance of the six vital signs respiratory rate, oxygen saturation, heart rate, systolic blood pressure, Glasgow coma scale (GCS) and temperature was included.

### Blood tests

Four blood tests were taken in the ambulance: P-Glucose, P-Lactate, P-Heparin-Binding Protein (HBP) and P-soluble urokinase Plasminogen Activator Receptor (suPAR). P-Glucose was chosen as it is included in a previous screening tool for sepsis for the ambulance [16], Lactate is a known marker for sepsis and mortality, included in several screening tools [16–18] and frequently used in Swedish EDs. HBP and suPAR were primarily included in the Predict Sepsis study due to promising results as biomarkers for sepsis at the start of the study [19, 20]. The blood tests were analysed by certified hospital- and university-bound laboratories after arrival to the ED. A detailed description of the handling and analyses of these blood tests is provided in the previously published original study by Wallgren et al [12].

### Demographic variables

Age, gender, and data required for the calculation of Charlson comorbidity score [19] were extracted from hospital records.

### Definitions

#### Infection

Infection was defined as infection according to the clinical judgment of the ambulance personnel and was documented in the study protocol. Ambulance personnel were blinded to results of the blood tests.

#### Mortality

Mortality was defined as in-hospital mortality, i.e., death during in-hospital care.

#### Sepsis

Sepsis was defined in accordance with the Sepsis-3 criteria [21], within 36 h from ED arrival [12], including criteria for infection, for details see Wallgren et al [12].

### Statistical analyses

Statistical analyses were performed using SPSS (Statistical Package for the Social Sciences) version 27.0 (SPSS Inc., Chicago, IL, USA).

Differences in proportions and medians between the groups with and without infection were analysed using Chi2 test and Mann–Whitney U test, respectively.

The association between the 21 variables and mortality was analysed using univariable regression analysis followed by multivariable logistic regression analysis including the variables which were associated significantly with mortality in the univariable analysis. Categorized variables were used for all regression analyses, with cut-offs calculated in the previous study [12]. Two-sided p-values were used. *P*-values < 0.05 were considered significant.

### Ethical approval

The study received approval from the Stockholm Regional Ethical Review Board (reference number 2016/2001–31/2, 2018/2202 and 2020–03,894). Written consent was obtained from all participants.

## Results

### Characteristics

**Fig. 1:**
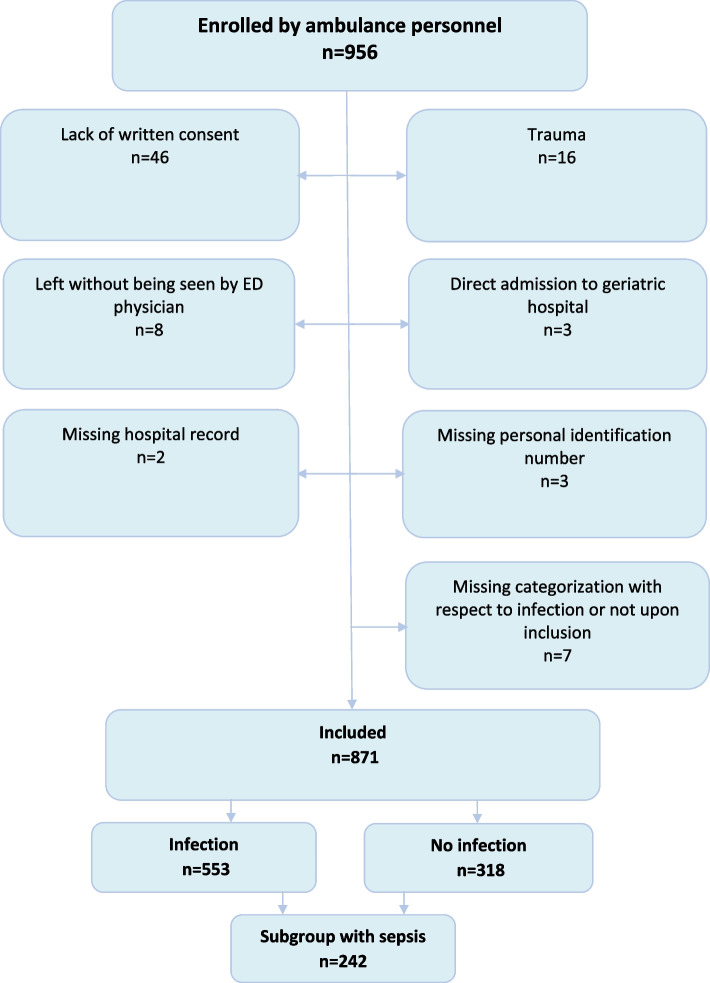
Flow chart of inclusion and exclusion. ED = Emergency Department

Eight hundred seventy-one patients were included in the ambulance, of which 553 were categorized as having an infection and 318 as having no infection, see Fig. 1. A total of 242 patients developed sepsis: 230/553 (41.6%) of those categorized as having an infection and 12/318 (3.8%) of those categorized as having no infection in the ambulance.

In total, 41 patients died during hospital stay: 33/553 (6.0%) with infection, 8/318 (2.5%) with no infection and 25/242 (10.0%) of the patients who developed sepsis. See Table 1 for characteristics of the study population.

### Association of variables measured in the ambulance and mortality

#### Patients with infection

An inability of the patient to answer questions relating to gastrointestinal symptoms or pain was significantly associated with mortality in univariable analysis, in addition to oxygen saturation < 94%, heart rate > 110 /min, GCS < 15, suPAR 4.0–7.9, suPAR ≥ 8.0 and Charlson comorbidity score ≥ 5, see Table 2.

**Table 2:** Association between 21 variables and in-hospital mortality among 553 ambulance patients with infection

Variable	Category	Crude		Univariable, unadjusted *n* = 553			Multivariable, adjusted** *n* = 514		
		***n* = 553**							
		n*	**% dead**	***P-value***	**OR**	**95%CI**	***P-value***	**OR**	**95%CI**
**Keywords related to medical history**									
Fever or suspected fever				0.384					
	yes	22/404	5.4	0.585	0.8	0.4–1.8	-	-	-
	no	9/134	6.7	Ref	Ref	Ref	-	Ref	-
	inability to answer	2/14	**14.3**	0.317	2.3	0.5–12.0	-	-	-
Pain				** < 0.001**			0.079		
	yes	9/256	3.5	0.157	0.6	0.2–1.3	0.367	0.7	0.3–1.6
	no	17/274	6.2	Ref	Ref	Ref	-	Ref	-
	inability to answer	7/21	**33.3**	**0.000**	7.6	2.7–21.2	0.059	5.4	0.9–31.5
Acute altered mental status				0.520					
	yes	18/328	5.5	0.713	0.9	0.4–1.8	-	-	-
	no	13/208	6.3	Ref	Ref	Ref	-	Ref	-
	inability to answer	2/16	**12.5**	0.346	2.1	0.4–10.4	-	-	-
Weakness of the legs				0.079					
	yes	24/420	5.7	0.646	1.3	0.5–3.4	-	-	-
	no	5/109	4.6	Ref	Ref	Ref	-	Ref	-
	inability to answer	4/23	**17.4**	**0.039**	4.4	1.1–17.8	-	-	-
Breathing difficulties				0.910					
	yes	18/280	6.4	0.681	1.2	0.6–2.4	-	-	-
	no	14/251	5.6	Ref	Ref	Ref	-	Ref	-
	inability to answer	1/19	5.3	0.954	0.9	0.1–7.6	-	-	-
Loss of energy				0.213					
	yes	27/491	5.5	0.471	0.7	0.2–2.0	-	-	-
	no	4/50	8.0	Ref	Ref	Ref	-	Ref	-
	inability to answer	2/11	**18.2**	0.318	2.6	0.4–16.1	-	-	-
Gastrointestinal symptoms				**0.025**			0.836		
	yes	11/188	5.9	0.694	1.2	0.5–2.6	0.558	1.3	0.6–3.0
	no	17/337	5.0	Ref	Ref	Ref	-	Ref	-
	inability to answer	5/26	**19.2**	**0.007**	4.5	1.5–13.3	0.822	1.2	0.2–7.4
Risk factors for sepsis				0.421					
	yes	15/229	6.6	0.481	1.3	0.6–2.7	-	-	-
	no	15/294	5.1	Ref	Ref	Ref	-	Ref	-
	inability to answer	3/27	**11.1**	0.206	2.3	0.6–8.6	-	-	-
**Vital signs**									
Respiratory rate, > 24 breaths/min									
	yes	14/189	7.4	0.317	1.4	0.7–2.9	-	-	-
	no	19/361	5.3	Ref	Ref	Ref	-	Ref	-
Oxygen saturation < 94%									
	yes	22/232	9.5	**0.005**	2.9	1.4–6.2	0.150	1.9	0.8–4.4
	no	11/319	3.4	Ref	Ref	Ref	-	Ref	-
Heart rate > 110 beats/min									
	yes	11/106	**10.4**	**0.038**	2.2	1.0–4.8	0.204	1.7	0.7–4.1
	no	22/446	4.9	Ref	Ref	Ref	-	Ref	-
Systolic blood pressure ≤ 100 mmHg									
	yes	6/54	**11.1**	0.103	2.2	0.9–5.5	-	-	-
	no	27/496	5.4	Ref	Ref	Ref	-	Ref	-
Level of consciousness, GCS < 15									
	yes	12/115	**10.4**	**0.021**	2.4	1.1–5.2	0.738	1.2	0.5–2.8
	no	19/416	4.6	Ref	Ref	Ref	-	Ref	-
Temperature, °C				0.135					
≤ 38.0	yes	18/235	7.7	Ref	Ref	Ref	-	Ref	-
38.1–38.5	yes	1/91	1.1	0.052	0.1	0.0–1.0	-	-	-
> 38.5	yes	13/223	5.8	0.437	0.7	0.4–1.6	-	-	-
**Blood tests**									
P-Glucose > 6.5 mmol/L									
	yes	25/410	6.1	0.571	0.8	0.3–1.8	-	-	-
	no	8/105	7.6	Ref	Ref	Ref	-	Ref	-
P-Lactate, mmol/L				0.165					-
≤ 2.0	yes	19/340	5.6	Ref	Ref	Ref	-	Ref	
2.1–4.0	yes	8/160	5.0	0.786	0.9	0.4–2.6	-	-	-
> 4.0	yes	5/38	**13.2**	0.079	2.6	0.9–7.3	-	-	-
P-suPAR, ng/mL				** < 0.001**			** < 0.001**		
< 4.0	yes	1/184	0.5	Ref	Ref	Ref	-	Ref	-
4.0–7.9	yes	14/263	5.3	**0.025**	10.3	1.3–78.9	0.088	6.1	0.8–49.0
≥ 8.0	yes	18/93	**19.4**	** < 0.001**	43.9	5.8–334.9	**0.002**	25.4	3.2–199.8
P-HBP ≥ 15.0 ng/mL									
	yes	20/235	8.5	0.060	2.0	1.0–4.1	-	-	-
	no	13/292	4.5	Ref	Ref	Ref	-	Ref	-
**Demographic variables**									
Age ≥ 65 years									
	yes	31/476	6.5	0.194	2.6	0.6–11.1	-	-	-
	no	2/77	2.6	Ref	Ref	Ref	-	Ref	-
Gender, male									
	yes	20/331	6.0	0.928	1.0	0.5–2.1	-	-	-
	no	13/222	5.9	Ref	Ref	Ref	-	Ref	-
Charlson comorbidity score ≥ 5 points									
	yes	11/87	**12.6**	**0.006**	2.9	1.4–6.3	0.110	2.0	0.9–4.7
	no	22/466	4.7	Ref	Ref	Ref	-	Ref	-

OR   Odds Ratio, CI = Confidence Interval, Ref = Reference, GCS = Glasgow Coma Scale, °C = degrees Celsius, suPAR = soluble urokinase Plasminogen Activating Receptor, HBP = Heparin Binding Protein

^*^of patients with documentation of the variable

^**^Adjusted for all factors that were significant in the univariable analysis

Bold numbers indicate an in-hospital mortality rate equal to or exceeding 10% and significant P-values, respectively

suPAR ≥ 8.0 remained significantly associated (*p*-value 0.002) in multivariable analysis (OR 25.4; 95% CI, 3.2–199.8), see Table 2.

#### Patients with no infection

suPAR ≥ 8.0 and a Charlson comorbidity score ≥ 5 were significantly associated with mortality in univariable analysis among ambulance patients without infection. suPAR ≥ 8.0 remained significantly associated (*p*-value 0.002) in the multivariable analysis (OR 56.1; 95% CI, 4.5–700.0), see Table 3.

**Table 3:** Association between 21 variables and in-hospital mortality among 318 ambulance patients without infection

**Variable**	**Category**	**Crude**		**Univariable, unadjusted**			Multivariable, adjusted**		
		***n* = 318**		***n* = 318**			***n* = 313**		
		n*	**% dead**	***P-value***	**OR**	**95%CI**	***P-value***	**OR**	**95%CI**
**Keywords related to medical history**									
Fever or suspected fever				0.972					
	yes	1/31	3.2	0.812	1.3	0.2–10.9	-	-	-
	no	7/279	2.5	Ref	Ref	Ref	-	Ref	-
	inability to answer	0/6	0.0	1.000	0.0	0.0-	-	-	-
Pain				0.881					
	yes	3/145	2.1	0.614	0.7	0.2–2.9	-	-	-
	no	5/168	3.0	Ref	Ref	Ref	-	Ref	-
	inability to answer	0/2	0.0	1.000	0.0	0.0-	-	-	-
Acute altered mental status				0.985					
	yes	2/70	2.9	0.860	1.2	0.2–5.9	-	-	-
	no	6/242	2.5	Ref	Ref	Ref	-	Ref	-
	inability to answer	0/4	0.0	1.000	0.0	0.0-	-	-	-
Weakness of the legs				0.631					
	yes	4/105	3.8	0.338	2.0	0.5–8.1	-	-	-
	no	4/205	2.0	Ref	Ref	Ref	-	Ref	-
	inability to answer	0/6	0.0	1.000	0.0	0.0-	-	-	-
Breathing difficulties				0.986					
	yes	2/70	2.9	0.868	1.1	0.2–5.8	-	-	-
	no	6/240	2.5	Ref	Ref	Ref	-	Ref	-
	inability to answer	0/5	0.0	1.000	0.0	0.0-	-	-	-
Loss of energy				0.457					
	yes	6/162	3.7	0.211	2.8	0.6–14.1	-	-	-
	no	2/148	1.4	Ref	Ref	Ref	-	Ref	-
	inability to answer	0/5	0.0	1.000	0.0	0.0-	-	-	-
Gastrointestinal symptoms				0.968					
	yes	2/67	3.0	0.798	1.2	0.2–6.3	-	-	-
	no	6/247	2.4	Ref	Ref	Ref	-	Ref	-
	inability to answer	0/2	0.0	1.000	0.0	0.0-	-	-	-
Risk factors for sepsis				0.986					
	yes	1/33	3.0	0.869	1.2	0.1–10.0	-	-	-
	no	7/275	2.5	Ref	Ref	Ref	-	Ref	-
	inability to answer	0/7	0.0	1.000	0.0	0.0-	-	-	-
**Vital signs**									
Respiratory rate > 24 breaths/min									
	yes	1/23	4.3	0.573	1.9	0.2–15.7	-	-	-
	no	7/292	2.4	Ref	Ref	Ref	-	Ref	-
Oxygen saturation < 94%									
	yes	2/34	5.9	0.207	2.9	0.6–14.8	-	-	-
	no	6/282	2.1	Ref	Ref	Ref	-	Ref	-
Heart rate > 110 beats/min									
	yes	1/29	3.4	0.743	1.4	0.2–12.0	-	-	-
	no	7/287	2.4	Ref	Ref	Ref	-	Ref	-
Systolic blood pressure ≤ 100 mmHg									
	yes	1/12	8.3	0.225	3.9	0.4–34.1	-	-	-
	no	7/304	2.3	Ref	Ref	Ref	-	Ref	-
Level of consciousness, GCS < 15									
	yes	0/19	0.0	1.000	0.0	0.0-	-	-	-
	no	8/289	2.8	Ref	Ref	Ref	-	Ref	-
Temperature, °C				1.000					
≤ 38.0	yes	8/312	2.6	Ref	Ref	Ref	-	Ref	-
38.1–38.5	yes	0/3	0.0	1.000	0.0	0.0-	-	-	-
> 38.5	yes	0/1	0.0	1.000	0.0	0.0-	-	-	-
**Blood tests**									
P-Glucose > 6.5 mmol/L									
	yes	7/188	3.7	1.000	62,476,921.4	0.0-	-	-	-
	no	0/108	0.0	Ref	Ref	Ref	-	Ref	-
P-Lactate, mmol/L				0.410					-
≤ 2.0	yes	3/206	1,5	Ref	Ref	Ref	-	Ref	
2.1–4.0	yes	3/87	3.4	0.286	2.4	0.5–12.2	-	-	-
> 4.0	yes	1/19	5.3	0.262	3.8	0.4–38.0	-	-	-
P-suPAR, ng/mL				** < 0.001**			**0.003**		
< 4.0	yes	1/192	0.5	Ref	Ref	Ref	-	Ref	-
4.0–7.9	yes	3/110	2.7	0.148	5.4	0.6–52.1	0.179	4.9	0.5–49.0
≥ 8.0	yes	3/11	**27.3**	** < 0.001**	71.6	6.7–767.1	**0.002**	56.1	4.5–700.0
P-HBP ≥ 15.0 ng/mL									
	yes	2/44	4.5	0.288	2.5	0.5–13.2	-	-	-
	no	5/265	1.9	Ref	Ref	Ref	-	Ref	-
**Demographic variables**									
Age ≥ 65 years									
	yes	7/220	3.2	0.281	3.2	0.4–26.3	-	-	-
	no	1/98	1.0	Ref	Ref	Ref	-	Ref	-
Gender, male									
	yes	4/169	2.4	0.857	0.9	0.2–3.6	-	-	-
	no	4/149	2.7	Ref	Ref	Ref	-	Ref	-
Charlson comorbidity score ≥ 5 points									
	yes	2/17	**11.8**	**0.028**	6.6	1.2–35.3	0.540	1.9	0.3–13.5
	no	6/301	2.0	Ref	Ref	Ref	-	Ref	-

OR = Odds Ratio, CI = Confidence Interval, Ref = Reference, GCS = Glasgow Coma Scale, °C = degrees Celsius, suPAR = soluble urokinase Plasminogen Activating Receptor, HBP = Heparin Binding Protein

^*^of patients with documentation of the variable

^**^Adjusted for all factors that were significant in the univariable analysis

Bold numbers indicate an in-hospital mortality rate equal to or exceeding 10% and significant P-values, respectively

#### Patients who developed sepsis

For the association between the 21 variables measured in the ambulance and mortality in patients who developed sepsis, see Table 4.

**Table 4:** Association between 21 variables and in-hospital mortality among 242 ambulance patients who developed sepsis within 36 h from ED arrival

Variable	Category	Crude		Univariable, unadjusted			Multivariable, adjusted**		
		***n* = 242**		***n* = 242**			***n* = 231**		
		n*	**% dead**	***P-value***	**OR**	**95%CI**	***P-value***	**OR**	**95%CI**
**Keywords related to medical history**									
Fever or suspected fever				0.507					
	yes	17/187	9.1	0.540	0.7	0.3–2.0	-	-	-
	no	6/49	**12.2**	Ref	Ref	Ref	-	Ref	-
	inability to answer	2/10	**20.0**	0.518	1.8	0.3–10.5	-	-	-
Pain				** < 0.001**			**0.006**		
	yes	7/108	6.5	0.434	0.7	0.3–1.8	0.487	0.7	0.2–2.1
	no	11/119	9.2	Ref	Ref	Ref	-	Ref	-
	inability to answer	7/17	**41.2**	**0.001**	7.5	2.3–24.1	**0.005**	13.2	2.2–78.9
Acute altered mental status				0.378					
	yes	14/166	8.4	0.227	0.6	0.2–1.4	-	-	-
	no	9/67	**13.4**	Ref	Ref	Ref	-	Ref	-
	inability to answer	2/13	**15.4**	0.798	1.2	0.2–6.6	-	-	-
Weakness of the legs				0.149					
	yes	18/186	9.7	0.631	1.4	0.4–4.9	-	-	-
	no	3/43	7.0	Ref	Ref	Ref	-	Ref	-
	inability to answer	4/17	**23.5**	0.084	4.2	0.8–21.6	-	-	-
Breathing difficulties				0.751					
	yes	16/139	**11.5**	0.451	1.4	0.6–3.4	-	-	-
	no	8/96	8.3	Ref	Ref	Ref	-	Ref	-
	inability to answer	1/10	**10.0**	0.793	1.3	0.1–12.1	-	-	-
Loss of energy				0.214					
	yes	21/223	9.4	0.701	0.7	0.2–3.5	-	-	-
	no	2/17	**11.8**	Ref	Ref	Ref	-	Ref	-
	inability to answer	2/6	**33.3**	0.276	3.5	0.4–33.3	-	-	-
Gastrointestinal symptoms				**0.044**			0.949		
	yes	9/100	9.0	0.891	1.1	0.4–2.7	0.747	1.2	0.4–3.4
	no	11/128	8.6	Ref	Ref	Ref	-	Ref	-
	inability to answer	5/18	**27.8**	**0.017**	4.4	1.3–14.8	0.995	1.0	0.2–6.4
Risk factors for sepsis				0.392					
	yes	10/112	8.9	0.834	0.9	0.4–2.2	-	-	-
	no	12/119	**10.1**	Ref	Ref	Ref	-	Ref	-
	inability to answer	3/14	**21.4**	0.216	2.4	0.6–10.0	-	-	-
**Vital signs**									
Respiratory rate > 24 breaths/min									
	yes	11/111	9.9	0.931	1.0	0.4–2.2	-	-	-
	no	14/134	**10.4**	Ref	Ref	Ref	-	Ref	-
Oxygen saturation < 94% %									
	yes	19/134	**14.2**	**0.026**	3.0	1.1–7.7	0.112	2.4	0.8–6.9
	no	6/110	5.5	Ref	Ref	Ref	-	Ref	-
Heart rate > 110 beats/min									
	yes	8/62	**12.9**	0.450	1.4	0.6–3.5	-	-	-
	no	17/183	9.3	Ref	Ref	Ref	-	Ref	-
Systolic blood pressure ≤ 100 mmHg									
	yes	5/38	**13.2**	0.548	1.4	0.5–3.9	-	-	-
	no	20/206	9.7	Ref	Ref	Ref	-	Ref	-
Level of consciousness, GCS < 15									
	yes	10/78	**12.8**	0.363	1.5	0.6–3.5	-	-	-
	no	14/157	8.9	Ref	Ref	Ref	-	Ref	-
Temperature, °C				0.210					
≤ 38.0	yes	13/75	**17.3**	Ref	Ref	Ref	-	Ref	-
38.1–38.5	yes	0/39	0.0	0.998	0.0	0.0-	-	-	-
> 38.5	yes	11/127	8.7	0.077	0.5	0.2–1.1	-	-	-
**Blood tests**									
P-Glucose > 6.5 mmol/L									
	yes	19/185	**10.3**	0.336	0.6	0.2–1.7	-	-	-
	no	6/38	**15.8**	Ref	Ref	Ref	-	Ref	-
P-Lactate, mmol/L				0.144					-
≤ 2.0	yes	11/130	8.5	Ref	Ref	Ref	-	Ref	
2.1–4.0	yes	6/79	7.6	0.820	0.9	0.3–2.5	-	-	-
> 4.0	yes	6/31	**19.4**	0.078	2.7	0.9–7.9	-	-	-
P-suPAR, ng/mL				** < 0.001**			** < 0.001**		
< 4.0	yes	1/62	1.6	Ref	Ref	Ref	-	Ref	-
4.0–7.9	yes	8/116	6.9	0.157	4.6	0.6–37.4	0.511	2.1	0.2–19.7
≥ 8.0	yes	15/61	**24.6**	**0.005**	19.6	2.5–153.6	**0.009**	16.1	2.0–128.6
P-HBP ≥ 15.0 ng/mL									
	yes	17/131	**13.0**	0.124	2.1	0.8–5.2	-	-	-
	no	7/103	6.8	Ref	Ref	Ref	-	Ref	-
**Demographic variables**									
Age ≥ 65 years									
	yes	22/208	**10.6**	0.631	1.4	0.4–4.8	-	-	-
	no	3/38	7.9	Ref	Ref	Ref	-	Ref	-
Gender, male									
	yes	16/156	**10.3**	0.968	1.0	0.4–2.4	-	-	-
	no	9/90	**10.0**	Ref	Ref	Ref	-	Ref	-
Charlson comorbidity score ≥ 5 points									
	yes	8/48	**16.7**	0.113	2.1	0.8–5.2	-	-	-
	no	17/198	8.6	Ref	Ref	Ref	-	Ref	-

ED = Emergency Department, OR = Odds Ratio, CI = Confidence Interval, Ref = Reference, GCS = Glasgow Coma Scale, °C = degrees Celsius, suPAR = soluble urokinase Plasminogen Activating Receptor, HBP = Heparin Binding Protein

^*^of patients with documentation of the variable

^**^Adjusted for all factors that were significant in the univariable analysis

Bold numbers indicate an in-hospital mortality rate equal to or exceeding 10% and significant P-values, respectively

Inability to answer questions relating to gastrointestinal symptoms or pain was significantly associated with mortality in univariable analysis, in addition to oxygen saturation < 94% and suPAR ≥ 8.0, see Table 4. Inability to answer questions relating to pain (OR 13.2; 95% CI, 2.2–78.9) and suPAR ≥ 8.0 (OR 16.1; 95% CI, 2.0–128.6) remained significantly associated in multivariable analysis (*p*-value 0.005 and *p*-value 0.009, respectively), see Table 4.

The mortality rate for septic patients with a body temperature of ≤ 38 °C was 17.3%, and for 38.0–38.5 °C it was 0% and > 38.5 it was 8.7%, *p*-value 0.007, see Table 4. Body temperature did not remain significant in the regression analyses, see Table 4.

## Discussion

SuPAR ≥ 8 ng/mL was the only variable which remained significantly associated with mortality among patients both with and without infection in the ambulance and in the subgroup who developed sepsis within 36 h from ED arrival. The highest mortality rate was observed among patients not able to answer questions relating to presenting symptoms and the mortality of these patients exceeded that of patients with severely deranged vital signs, both among patients with infection and among patients who developed sepsis. Septic patients with a normal body temperature had a higher mortality as compared to those with fever. Moreover, septic patients denying pain had a higher mortality as compared with those affirming pain. However, these latter two observations did not remain significant in the regression analysis. Identification of patients at risk of poor outcome in the ambulance could enable a higher triage level and a different approach with respect to subsequent monitoring, diagnostic work-up and treatment.

### suPAR

suPAR ≥ 8.0 ng/mL the only one of the 21 variables that remained significantly associated with mortality in the multivariable analysis among patients both with and without an infection, and among those with sepsis. suPAR is the soluble form of the membrane bound protein urokinase plasminogen activator receptor, present on immunologically active cells such as neutrophils. Elevated suPAR levels indicate activation of the immune system and has been suggested to represent inflammation at the cellular level in contrast to C-reactive Protein (CRP) that has been suggested to represent inflammation at a metabolic level [22, 23]. suPAR has been shown to be a prognostic marker in a wide range of conditions involving an inflammatory state, including sepsis and pneumonia but also myocardial infarction, chronic obstructive pulmonary disease (COPD), chronic kidney disease, diabetes, and cancer [19, 24–30] and suPAR is measured routinely in some EDs in Denmark [31]. Levels < 4.0 have been considered as “low risk” among ED patients and support discharge from the ED [32], while levels > 6.0 ng/mL have been proposed to indicate a high risk for death [32]. The current results support that high levels of suPAR are associated with a poor outcome not only among patients with an infection.

### The inability of a patient to answer questions relating to presenting symptoms

We believe that the inability of the patient to answer questions relating to symptoms is reflective of an altered mental status, and possibly a reduced sensory perception due to an affected general condition. Interestingly, the inability of patients with infection to answer questions relating to gastrointestinal symptoms, pain and weakness of the legs was more important for the mortality rate than a decreased level of consciousness and severely deranged vital signs. We speculate that it may be more difficult for a bystander to observe and add information concerning gastrointestinal symptoms or pain, as compared to e.g. acute altered mental status and fever. This could potentially explain the observed difference in mortality depending on which symptom the patient was unable to answer. These results are to our knowledge novel and should be further investigated in studies that do not include information of the medical history from bystanders. Inability to answer questions relating to presenting symptoms may be an expression of an altered mental status and could potentially add complementary information to e.g. the GCS as it seems to better relate to outcome.

### Fever

Body temperature showed an interesting relationship with mortality among septic patients, in that patients with a moderate fever appeared to have the lowest mortality rate. However, this observation did not remain significant in the regression analyses. That septic patients presenting with fever have a better prognosis has previously been presented by Sunden-Cullberg et al. [33], and that patients with fever receive a more timely and better quality of care [33]. Interestingly, they could also show that the lower mortality was not attributable to the improved care [33], raising the question of whether fever is protective per se. Several studies support fever being protective [34–36] and the suggested mechanistic explanations are e.g. direct killing of pathogens, induction of cytoprotective proteins in host cells in turn increasing the killing of pathogens, and activation of the immune system [34–36]. Our results support that the lack of fever signals a patient whose immune system is not responding appropriately to an infection, thus indicating the risk of a poor outcome.

### Pain

Patients with infection and sepsis that confirmed having pain had a significantly lower mortality, as compared to those denying pain. However, this finding did not remain significant in the regression analyses (Tables 2, and 4). Pain could signal the origin of an infection, which may enable a more directed treatment, in turn reducing mortality. We speculate that the denial of pain could possibly reflect a reduced sensory perception due to an affected general condition. Interestingly, this does not appear to be associated with a decreased level of consciousness, as discussed above.

### Limitations

This is, to the best of our knowledge, the first prospective study in the ambulance setting evaluating the association between ambulance variables and mortality among patients with and without infection.

The major limitation was the size of the study population. The current study was part of the Predict Sepsis study [12] and its power calculation was performed to include a sufficient number of patients with the outcome sepsis. Therefore, the study population is likely to be underpowered with respect to analyses of mortality which we expect led to non-significant association for previously described risk factors for death such as age and comorbidity. The cut-off for suPAR applied in the current study (≥ 8.0) was chosen based on the strongest association to sepsis in the study of sepsis identification [12] and hence not calculated for the outcome mortality. Cut-offs calculated based on the strongest association with mortality should be used in future studies. Nevertheless, our results support that elevated levels of suPAR are associated with a poor outcome in patients presenting to the ambulance both with and without infection. It is, however, in this context important to point out that the patients with no infection in the ambulance are unwell and therefore likely to have ongoing inflammatory processes which is picked up by an elevated suPAR. Furthermore, our results suggest that patients with infection not able to answer questions relating to presenting symptoms are at risk of poor outcome, which is novel and requires further investigation. Finally, to define infection based on clinical judgment can be questioned as it may lend to subjectivity and affect the reproducibility of the study. However, there was a high degree of agreement between ambulance personnel clinical judgment of suspected infection and the previously developed criteria-based definition of infection [12], supporting the ambulance personnel accurate clinical judgment of infection.

## Conclusions

suPAR ≥ 8.0 ng/mL was associated with mortality in patients presenting to the ambulance both with and without an infection, and among patients who developed sepsis within 36 h from ED arrival. In addition, the results indicate that the inability of an ambulance patient with an infection to answer questions relating to presenting symptoms is associated with a high mortality which exceeds that of both a decreased level of consciousness and severely deviated vital signs. These results suggest that suPAR and medical history are valuable tools with which to identify patients at risk of poor outcome in the ambulance and could potentially signal the need of enhanced attention.

## Data Availability

The data that support the findings of this study are available from Karolinska Institutet Södersjukhuset and Örebro University, but restrictions apply to the availability of these data, which were used under license for the current study, and so are not publicly available. Data are however available from the authors upon reasonable request and with permission of Karolinska Institutet Södersjukhuset and Örebro University.

## Abbreviations

GCS: Glasgow Coma Scale
suPAR: Soluble urokinase Plasminogen Activator Receptor
ng/mL: Nanograms per milliliter
OR: Odds Ratio
CI: Confidence Interval
NCT: National Clinical Trial
ED: Emergency Department
HBP: Heparin-Binding Protein
SOFA: Sequential Organ Failure Assessment
SPSS: Statistical Package for the Social Sciences
CRP: C-reactive Protein
COPD: Chronic Obstructive Pulmonary Disease
IQR: Interquartile Range
